# Supplementary material for: Do Patient-Reported Outcome Measures (PROMs) Used Within Radiotherapy Clinical Trials Reflect the Impact of Treatment?
Source: Cancers (Basel). 2024 Nov 14;16(22):3832. doi: 10.3390/cancers16223832 (PMC11592418; doi:10.3390/cancers16223832)
Supplement: Supplementary file 1 [file cancers-16-03832-s001.zip › cancers-3305883-supplementary.pdf]

Supplementary file/Table S1 – PROM data extraction from identified articles.

Name/Year	Title	Geographical location	Study population	PROM included	RT or PBT
Ahmed et al. 2021	Patient-reported functional outcomes in a cohort of hand and foot sarcoma survivors treated with limb sparing surgery and radiation therapy	USA	Sarcoma	TESS-UE, TESS-LE, MHQ, FAOS	RT
Almstahl et al. 2016	Explorative study on quality of life in relation to salivary secretion rate in patients with head and neck cancer treated with radiotherapy	Sweden	Head and neck	EORTC-QLQ-C30, EORTC-QLQ-HN35, HADS	RT
Almstahl et al. 2019	Explorative study on quality of life in relation to salivary secretion rate in head and neck cancer patients treated with radiotherapy up to 2 years post treatment	Sweden	Head and neck	EORTC-QLQ-C30, EORTC-QLQ-HN35, HADS	RT
Aoki et al. 2023	Autonomic function measurements for evaluating fatigue and quality of life in patients with breast cancer undergoing radiation therapy: a prospective longitudinal study	Japan	Breast	Cancer Fatigue Scale, SF-8	RT
Bagley et al. 2023	Xerostomia-related quality of life for patients with oropharyngeal carcinoma treated with proton therapy	USA	Head and neck	XeQoLS	PBT
Bai et al. 2020	Comparing bowel and urinary domains of patient-reported quality of life at the end of and 3 months post radiotherapy between intensity-modulated radiotherapy and proton beam therapy for clinically localized prostate cancer	USA	Prostate	EPIC-26	RT and PBT
Bandarage et al. 2016	Prospective evaluation of patient-reported quality of life outcomes after external beam radiation treatment for prostate cancer in Victoria: A cohort study by the Victorian Prostate Cancer Registry	Australia	Prostate	EPIC-26	RT
Baviskar et al. 2023	Short-course Palliative Hypofractionated Radiotherapy in Patients with Poor-prognosis High-grade Glioma: Survival and Quality of Life Outcomes from a Prospective Phase II Study	India	Brain	EORTC-QLQ-C30, EORTC-QLQ-BN20	RT
Belkacemi et al. 2023	Effect of Serelys Homme on the Incidence and Severity of Vasomotor Symptoms and Quality-of-Life Impairments in Patients Receiving Hormone Therapy and Radiation for Localized Prostate Cancer: Results of the ESCULAPE Phase 2 Prospective Study	France	Prostate	EQ-5D-5L, FACT-P, HFR-DIS	RT
Bhattacharya et al. 2019	Patient-reported outcomes over 5 years after whole- Or partial-breast radiotherapy: Longitudinal analysis of the import low (CRUK/ 06/003) phase III randomized controlled trial	UK	Breast	EORTC-QLQ-C30, EORTC-QLQ-BR23, Body image scale, HADS	RT
Brundage et al. 2015	Impact of radiotherapy when added to androgen-deprivation therapy for locally advanced prostate cancer: Long-term quality-of-life outcomes from the NCIC CTG PR3/MRC PR07 randomized trial	North America	Prostate	EORTC-QLQ-C30, EORTC-QLQ-PR19, FACT-P	RT
Chera et al. 2018	Mature results of a prospective study of deintensified chemoradiotherapy for low-risk human papillomavirus-associated oropharyngeal squamous cell carcinoma	USA	Head and neck	PRO-CTCAE	RT
Choudhury et al. 2024	Timing of High-Dose-Rate Brachytherapy With External Beam Radiation Therapy in Patients With Intermediate- and High-Risk Localized Prostate Cancer and Its Effects on Toxicity and Quality of Life: A Randomized Controlled Trial (THEPCA)	UK	Prostate	IPSS, IIEF-5, FACT-P	RT

Cloitre et al. 2023	Toxicity, quality of life, and PSA control after 50 Gy stereotactic body radiation therapy to the dominant intraprostatic nodule with the use of a rectal spacer: results of a phase I/II study	Switzerland	Prostate	IPSS, EORTC-QLQ-PR25	RT
Crook et al. 2024	A Randomized Trial Comparing Quality of Life After Low-Dose Rate or High-Dose Rate Prostate Brachytherapy Boost With Pelvic External Beam Radiation Therapy	Canada	Prostate	EPIC, IPSS, IIEF-5	RT
de Vasconcellos Ferreira et al. 2023	Evaluation of oral mucositis, candidiasis, and quality of life in patients with head and neck cancer treated with a hypofractionated or conventional radiotherapy protocol: a longitudinal, prospective, observational study	Brazil	Head and neck	EORTC-QLQ-C30, EORTC-QLQ-HN35	RT
Dixon et al. 2018	Long term patient reported swallowing function following chemoradiotherapy for oropharyngeal carcinoma	UK	Head and neck	MDADI	RT
Ferrario et al. 2024	Toxicity profile and Patient-Reported outcomes following salvage Stereotactic Ablative Radiation Therapy to the prostate Bed: The POPART multicentric prospective study	Italy	Prostate	EPIC, IIEF-5, ICIQ-UI SF	RT
Garden et al. 2020	Neurologic sequelae following radiation with and without chemotherapy for oropharyngeal cancer: Patient reported outcomes study	USA	Head and neck	MDASI-HN	RT
Ghadjar et al. 2015	Acute Toxicity and Quality of Life After Dose-Intensified Salvage Radiation Therapy for Biochemically Recurrent Prostate Cancer After Prostatectomy: First Results of the Randomized Trial SAKK 09/10	Switzerland	Prostate	EORTC-QLQ-C30, EORTC-QLQ-PR25	RT
Gil et al. 2023	A phase II randomized clinical trial to assess toxicity and quality of life of breast cancer patients with hypofractionated versus conventional fractionation radiotherapy with regional nodal irradiation in the context of COVID-19 crisis	Brazil	Breast	EORTC-QLQ-C30, EORTC-QLQ-BR23	
Habets et al. 2016	Neurocognitive functioning and health-related quality of life in patients treated with stereotactic radiotherapy for brain metastases: a prospective study	Netherlands	Brain	EORTC-QLQ-C30, EORTC-QLQ-BN20	RT
Haussmann et al. 2023	Comparison of adverse events in partial- or whole breast radiotherapy: investigation of cosmesis, toxicities and quality of life in a meta-analysis of randomized trials	Germany	Breast	EORTC-QLQ-C30, EORTC-QLQ-BR23	RT
Heggebo et al. 2023	Investigating survival, quality of life and cognition in PROton versus photon therapy for IDH- mutated diffuse grade 2 and 3 GLIOmas (PRO-GLIO): a randomised controlled trial in Norway and Sweden	Norway	Brain	EORTC-QLQ-C30, EORTC-QLQ-BN20, EQ-5D-5L, Chalder Fatigue Questionnaire, GAD-7, PHQ-9	PBT and RT
Hoffman et al. 2018	Patient-reported Urinary, Bowel, and Sexual Function After Hypofractionated Intensity-modulated Radiation Therapy for Prostate Cancer: Results From a Randomized Trial	USA	Prostate	Self-developed	RT
Hoppe et al. 2023	Pragmatic, Prospective Comparative Effectiveness Trial of Carbon Ion Therapy, Surgery, and Proton Therapy for the Management of Pelvic Sarcomas (Soft Tissue/Bone) Involving the Bone: The PROSPER Study Rationale and Design	USA	Sarcoma	PROMIS-29, EORTC-QLQ-CR29	PBT
Hopwood et al. 2010	Comparison of patient-reported breast, arm, and shoulder symptoms and body image after radiotherapy for early breast cancer: 5-year	UK	Breast	EORTC-QLQ-C30, EORTC-QLQ-BR23, self-developed questionnaire from IMPORT-LOW	RT

	follow-up in the randomised Standardisation of Breast Radiotherapy (START) trials				
Hsu et al. 2016	Efficacy of Traditional Chinese Medicine in Xerostomia and Quality of Life during Radiotherapy for Head and Neck Cancer: A Prospective Pilot Study	Taiwan	Head and neck	EORTC-QLQ-HN35	RT
Huang et al. 2018	The effectiveness of a saline mouth rinse regimen and education programme on radiation-induced oral mucositis and quality of life in oral cavity cancer patients: A randomised controlled trial	Taiwan	Head and neck	UW-QOL	RT
Iarrobino et al. 2019	Early Exploratory Analysis for Patient-reported Quality of Life and Dosimetric Correlates in Hypofractionated Stereotactic Body Radiation Therapy (SBRT) for Low-risk and Intermediate-risk Prostate Cancer: Interim Results from a Prospective Phase II Clinical Trial	USA	Prostate	EPIC-26	RT
Jain et al. 2023	Long-Term Yogic Intervention Improves Symptomatic Scale and Quality of Life by Reducing Inflammatory Cytokines and Oxidative Stress in Breast Cancer Patients Undergoing Chemotherapy and/or Radiotherapy: A Randomized Control Study	India	Breast	EORTC-QLQ-C30	RT
Kan et al. 2023	The quality of life in nasopharyngeal carcinoma radiotherapy: A longitudinal study	China	Head and neck	EORTC-QLQ-C30	RT
Kangas et al. 2012	The effects of radiotherapy on psychosocial and cognitive functioning in adults with a primary brain tumor: a prospective evaluation	Australia	Brain	FACT-Br	RT
Katzel et al. 2023	Real-World Use of Electronic Patient-Reported Outcome (ePRO) Tools Integrated in the Electronic Medical Record During Radiation Therapy for Head and Neck Cancer: Feasibility Study	US	Head and neck	FACT-HN	RT
Kosgallana et al. 2023	Oral health related quality of life of oral cancer patients treated with radiotherapy alone or with chemotherapy in a tertiary referral centre in Sri Lanka	Sri Lanka	Head and neck	EORTC-QLQ-OH15	RT
Kumarasiri et al. 2024	Radiation therapy margin reduction for patients with localized prostate cancer: A prospective study of the dosimetric impact and quality of life	US	Prostate	EPIC-26	RT
Lagerwaard et al. 2023	Patient-reported quality of life after stereotactic ablative radiotherapy for early-stage lung cancer	Netherlands	Lung	EORTC-QLQ-C30	RT
Laughlin et al. 2023	Initial Quality of Life and Toxicity Analysis of a Randomized Phase 3 Study of Moderately Hypofractionated Radiation Therapy With or Without Androgen Suppression for Intermediate-Risk Adenocarcinoma of the Prostate: PCG GU003	US	Prostate	EPIC, SF-12 and IPSS	PBT
Lee et al. 2011	Effects of a nurse-led cognitive-behaviour therapy on fatigue and quality of life of patients with breast cancer undergoing radiotherapy: an exploratory study	Korea	Breast	Revised Piper Fatigue Scale, C-QOL	RT
Lee et al. 2021	The effect of comprehensive oral care program on oral health and quality of life in patients undergoing radiotherapy for head and neck cancer: A quasi-experimental case-control study	Korea	Head and neck	EORTC-QLQ-HN35	RT

Lim et al. 2021	Can the Risk of Dysphagia in Head and Neck Radiation Therapy Be Predicted by an Automated Transit Fluence Monitoring Process During Treatment? A First Comparative Study of Patient Reported Quality of Life and the Fluence-Based Decision Support Metric	USA	Head and neck	MDADI	RT
Lombardi et al. 2018	Quality of Life Perception, Cognitive Function, and Psychological Status in a Real-world Population of Glioblastoma Patients Treated With Radiotherapy and Temozolomide: A Single-center Prospective Study	Italy	Brain	EORTC-QLQ-C30, EORTC-QLQ-BN20, HADS	RT
Ma et al. 2023	Quality-of-Life Outcomes and Toxicity Profile Among Patients With Localized Prostate Cancer After Radical Prostatectomy Treated With Stereotactic Body Radiation: The SCIMITAR Multicenter Phase 2 Trial	US	Prostate	EPIC-26, IPSS	RT
Martins et al. 2023	Effects of a mucoadhesive phytomedicine (Curcuma longa L. and Bidens pilosa L.) on radiotherapy-induced oral mucositis and quality of life of patients undergoing head and neck cancer treatment: randomized clinical trial	Brazil	Head and neck	OHIP-14, PROMS scale	RT
Matsukawa et al. 2020	Health-related quality of life in Japanese patients with prostate cancer following proton beam therapy: an institutional cohort study	Japan	Prostate	EPIC	PBT
Moinpour et al. 2008	Health-related quality of life results in pathologic stage C prostate cancer from a Southwest Oncology Group trial comparing radical prostatectomy alone with radical prostatectomy plus radiation therapy	USA	Prostate	SWOG-QOL	RT
Morelli et al. 2023	Impact of radiation dose on patient-reported acute taste alteration in a prospective observational study cohort in head and neck squamous cell cancer (HNSCC)	Italy	Head and neck	EORTC-QLQ-C30, EORTC-QLQ-HN35	RT
Movsas et al. 2023	Dose-Escalated Radiation Alone or in Combination with Short-Term Total Androgen Suppression for Intermediate-Risk Prostate Cancer: Patient-Reported Outcomes from NRG/Radiation Therapy Oncology Group 0815 Randomized Trial	US	Prostate	EPIC, PROMIS-29, EQ-5D-5L	RT
Nakano et al. 2020	The neurocognitive function change criteria after whole-brain radiation therapy for brain metastasis, in reference to health-related quality of life changes: A prospective observation study	Japan	Brain	EORTC-QLQ-C30, EORTC-QLQ-BN20	RT
Ng et al. 2020	Patient-reported outcomes after surgery or radiotherapy for localised prostate cancer: a retrospective study	Hong Kong	Prostate	EPIC	RT
Nicolaisen et al. 2014	Quality of life and satisfaction with information after radical prostatectomy, radical external beam radiotherapy and postoperative radiotherapy: a long-term follow-up study	Norway	Prostate	SF-12, EPIC	RT
Nossiter et al. 2020	Patient-Reported Functional Outcomes After Hypofractionated or Conventionally Fractionated Radiation for Prostate Cancer: A National Cohort Study in England	UK	Prostate	EPIC-26, EQ-5D-5L	RT
Oskam et al. 2010	Quality of life as predictor of survival: a prospective study on patients treated with combined surgery and radiotherapy for advanced oral and oropharyngeal cancer	Netherlands	Head and neck	EORTC-QLQ-C30	RT
Ozgen et al. 2023	Radiation pneumonitis in relation to pulmonary function, dosimetric factors, TGFbeta1 expression, and quality of life in breast cancer patients receiving post-operative radiotherapy: a prospective 6-month follow-up study	Turkey	Breast	EORTC-QLQ-C30, EORTC-QLQ-BR23	

Parry et al. 2020	Toxicity of Pelvic Lymph Node Irradiation With Intensity Modulated Radiation Therapy for High-Risk and Locally Advanced Prostate Cancer: A National Population-Based Study Using Patient-Reported Outcomes	UK	Prostate	EPIC-26, EQ-5D-5L	RT
Parsai et al. 2020	Heterogenous Dose-escalated Prostate Stereotactic Body Radiation Therapy for All Risk Prostate Cancer: Quality of Life and Clinical Outcomes of an Institutional Pilot Study	USA	Prostate	EPIC-26	RT
Pasquier et al. 2019	Intensity-modulated radiation therapy with simultaneous integrated boost for locally advanced breast cancer: a prospective study on toxicity and quality of life	France	Breast	EORTC-QLQ-C30, EORTC-QLQ-BR23	RT
Petersson et al. 2023	A randomized controlled study evaluating the head-lift exercise in head and neck cancer patients with radiation-induced dysphagia: effect on swallowing function and health-related quality of life over 12 months	Sweden	Head and neck	MDADI, EORTC-QLQ-C30, EORTC-QLQ-HN35	RT
Ringash et al. 2017	Quality of life and swallowing with standard chemoradiotherapy versus accelerated radiotherapy and panitumumab in locoregionally advanced carcinoma of the head and neck: A phase III randomised trial from the Canadian Cancer Trials Group (HN.6)	Canada	Head and neck	FACT-HN, MDADI, SWAL-QOL	RT
Sahoo et al. 2023	A Prospective Cohort Study Analyzing Radiation-Induced Xerostomia and Quality of Life of Head and Neck Cancer Patients Treated With Intensity-Modulated Radiotherapy and 3D Conformal Radiotherapy Techniques at a Tertiary Cancer Center in Eastern India	India	Head and neck	XeQoLS	RT
Schad et al. 2023	Evaluation of quality of life in lung cancer patients receiving radiation and Viscum album L.: a real-world data study	Germany	Lung	EORTC-QLQ-C30	RT
Sher et al. 2023	Efficacy and Quality-of-Life Following Involved Nodal Radiotherapy for Head and Neck Squamous Cell Carcinoma: The INRT-AIR Phase II Clinical Trial	US	Head and neck	MDADI	RT
Singer et al. 2023	The health-related quality of life of sarcoma patients treated with neoadjuvant versus adjuvant radiotherapy - Results of a multi-center observational study	Germany	Sarcoma	EORTC-QLQ-C30	RT
Slotman et al. 2009	Prophylactic cranial irradiation in extensive disease small-cell lung cancer: short-term health-related quality of life and patient reported symptoms: results of an international Phase III randomized controlled trial by the EORTC Radiation Oncology and Lung Cancer Groups	Netherlands	Brain (Lung primary)	EORTC-QLQ-C30, EORTC-QLQ-BN20	RT
Soffietti et al. 2013	A European Organisation for Research and Treatment of Cancer phase III trial of adjuvant whole-brain radiotherapy versus observation in patients with one to three brain metastases from solid tumors after surgical resection or radiosurgery: quality-of-life results	Italy	Brain	EORTC-QLQ-C30, EORTC-QLQ-BN20	RT
Stewart et al. 2018	Evaluating quality of life and cost implications of prophylactic radiotherapy in mesothelioma: Health economic analysis of the SMART trial	UK	Lung	EORTC-QLQ-C30, EQ-5D-5L	RT
Takizawa et al. 2009	Oncological results, functional outcomes and health-related quality-of-life in men who received a radical prostatectomy or external beam radiation therapy for localized prostate cancer: a study on long-term patient outcome with risk stratification	Japan	Prostate	SF-36, UCLA PCI	RT

Tan et al. 2022	Efficacy, toxicity, and quality-of-life outcomes of ultrahypofractionated radiotherapy in patients with localized prostate cancer: A single-arm phase 2 trial from Asia	Singapore	Prostate	EPIC	RT
Taphoorn et al. 2015	Health-Related Quality of Life in a Randomized Phase III Study of Bevacizumab, Temozolomide, and Radiotherapy in Newly Diagnosed Glioblastoma	Global	Brain	EORTC-QLQ-C30, EORTC-QLQ-BN20	RT
Tuomi et al. 2021	Voice Quality, Function, and Quality of Life for Laryngeal Cancer: A Prospective Longitudinal Study Up to 24 Months Following Radiotherapy	Sweden	Head and neck	EORTC-QLQ-C30, EORTC-QLQ-HN35, S-SECEL	RT
Ursino et al. 2023	Patient-Reported Outcomes After Swallowing (SWOARs)-Sparing IMRT in Head and Neck Cancers: Primary Results from a Prospective Study Endorsed by the Head and Neck Study Group (HNSG) of the Italian Association of Radiotherapy and Clinical Oncology (AIRO)	Italy	Head and neck	MDADI	RT
Parijs et al. 2021	Cardiopulmonary-related patient-reported outcomes in a randomized clinical trial of radiation therapy for breast cancer	Belgium	Breast	EORTC-QLQ-C30	RT
Van Aperen et al. 2023	EffEx-HN trial: study protocol for a randomized controlled trial on the EFFectiveness and feasibility of a comprehensive supervised EXercise program during radiotherapy in Head and Neck cancer patients on health-related quality of life	Belgium	Head and neck	EORTC-QLQ-C30	RT
van der Weijst et al. 2023	Patient-reported outcomes and functional exercise capacity in a real-life setting in non-small cell lung cancer patients undergoing stereotactic body radiotherapy: the Lung PLUS study	Belgium	Lung	PRO-CTCAE, EORTC-QLQ-C30, EORTC-QLQ-LC13	RT
Velikova et al. 2018	Quality of life after postmastectomy radiotherapy in patients with intermediate-risk breast cancer (SUPREMO): 2-year follow-up results of a randomised controlled trial	UK	Breast	EORTC-QLQ-C30, EORTC-QLQ-BR23, Body image scale, HADS, EQ-5D-3L	RT
Versmessen et al. 2012	Health-related quality of life in survivors of stage I-II breast cancer: randomized trial of post-operative conventional radiotherapy and hypofractionated tomotherapy	Belgium	Breast	EORTC-QLQ-C30, EORTC-QLQ-BR23	RT
Wan Leung et al. 2011	Health-related quality of life in 640 head and neck cancer survivors after radiotherapy using EORTC QLQ-C30 and QLQ-H&N35 questionnaires	Taiwan	Head and neck	EORTC-QLQ-C30, EORTC-QLQ-HN35	RT
Wang et al. 2009	Cognition and quality of life after chemotherapy plus radiotherapy (RT) vs. RT for pure and mixed anaplastic oligodendrogliomas: radiation therapy oncology group trial 9402	USA	Brain	EORTC-QLQ-C30, EORTC-QLQ-BN20	RT
Weiss et al. 2023	The impact of palliative radiotherapy on health-related quality of life in patients with head and neck cancer - Results of a multicenter prospective cohort study	Germany	Prostate	EORTC-QLQ-C30, EORTC-QLQ-HN35	RT
Wilkins et al. 2015	Hypofractionated radiotherapy versus conventionally fractionated radiotherapy for patients with intermediate-risk localised prostate cancer: 2-year patient-reported outcomes of the randomised, non-inferiority, phase 3 CHHiP trial	UK	Prostate	UCLA-PCI, EPIC-26, EPIC (Full), SF-36, SF-12, FACT-P	RT
Wong et al. 2017	Long-Term Quality of Life of Retroperitoneal Sarcoma Patients Treated with Pre-Operative Radiotherapy and Surgery	Canada	Sarcoma	EORTC-QLQ-C30	RT
Xiao et al. 2017	Quality of Life and Performance Status From a Substudy Conducted Within a Prospective Phase 3 Randomized Trial of Concurrent Standard Radiation Versus Accelerated Radiation Plus Cisplatin for Locally Advanced Head and Neck Carcinoma: NRG Oncology RTOG 0129	USA	Head and Neck	HNRQ, SQLI	RT

Zetner et al. 2023	Quality-of-life outcomes following topical melatonin application against acute radiation dermatitis in patients with early breast cancer: A double-blind, randomized, placebo-controlled trial	Denmark	Breast	EORTC-QLQ-C30, EORTC-QLQ-BR23	RT

BN—Brain module, BR- Breast module, C30—Core questionnaire, C-QOL - quality of life scale for Korean patients with cancer, EORTC—European Organisation for Research and Treatment of Cancer, EPIC—Expanded Prostate cancer Index Composite, EQ-5D—EuroQoL 5 level questionnaire, FACT—Functional Assessment of Cancer Therapy, FAOS—Foot and Ankle Outcome Score, GAD-7—Generalised Anxiety Disorder questionnaire, HADS—Hospital Anxiety and Depression Scale, HFR-DIS—Hot Flash Related Daily Interference Scale, HN—Head and Neck module, HNRQ—Head and Neck Radiotherapy Questionnaire, ICIQ-UI SF - International Consultation on Incontinence Questionnaire-Urinary Incontinence Short Form, IIEF-5—International Index of Erectile Function, IPSS—International Prostate Symptom Scale, LC—Lung cancer module, MDADI—MD Anderson Dysphagia Inventory, MDASI-HN—MD Anderson Symptom Inventory for Head and Neck cancer, MHQ—Michigan Hand outcomes Questionnaire, OHIP-14—Oral Health Impact Profile-14, PHQ-9—Patient Health Questionnaire, P—prostate module, PR—prostate module, PRO-CTCAE - patient reported outcome version of the Common Terminology Criteria for Adverse Events, PROM—patient-reported outcome measure, PROMS scale—Patient Reported Oral Mucositis Symptom scale, S-SECEL—Swedish Self Evaluation of Communication Experiences after Laryngeal problems, SF—Short Form survey, SQLI—Spitzer Quality of Life Index, SWAL-QOL—Swallowing Quality of Life Questionnaire, SWOG-QoL—the South West Oncology Group Quality of Life questionnaire, TESS-LE—Toronto Extremity Salvage Score Lower Extremity, TESS-UE—Toronto Extremity Salvage Score Upper Extremity, UCLA-PCI—UCLA Prostate Cancer Index, UW-QOL—University of Washington Quality of Life Questionnaire, XeQoLS—Xerostomia related Quality of Life Scale.
